# Elastic Kernmantle E‐Braids for High‐Impact Sports Monitoring

**DOI:** 10.1002/advs.202202489

**Published:** 2022-06-27

**Authors:** Wei Wang, Aifang Yu, Yulong Wang, Mengmeng Jia, Pengwen Guo, Lele Ren, Di Guo, Xiong Pu, Zhong Lin Wang, Junyi Zhai

**Affiliations:** ^1^ CAS Center for Excellence in Nanoscience Beijing Key Laboratory of Micro‐nano Energy and Sensor Beijing Institute of Nanoenergy and Nanosystems Chinese Academy of Sciences Beijing 101400 China; ^2^ School of Nanoscience and Technology University of Chinese Academy of Sciences Beijing 100049 P. R. China; ^3^ Center on Nanoenergy Research School of Physical Science and Technology Guangxi University Nanning 530004 P. R. China; ^4^ School of Materials Science and Engineering Georgia Institute of Technology Atlanta GA 30332 USA

**Keywords:** E‐braids, flexible electronics, intelligent sports, kernmantle, triboelectric nanogenerator

## Abstract

The kernmantle construction, a kind of braiding structure that is characterized by the kern absorbing most of the stress and the mantle protecting the kern, is widely employed in the field of loading and rescue services, but rarely in flexible electronics. Here, a novel kernmantle electronic braid (E‐braid) for high‐impact sports monitoring, is proposed. The as‐fabricated E‐braids not only demonstrate high strength (31 Mpa), customized elasticity, and nice machine washability (>500 washes) but also exhibit excellent electrical stability (>200 000 cycles) during stretching. For demonstration, the E‐braids are mounted to different parts of the trampoline for athletes’ locomotor behavior monitoring. Furthermore, the E‐braids are proved to act as multifarious intelligent sports gear or wearable equipment such as electronic jump rope and respiration monitoring belt. This study expands the kernmantle structure to soft flexible electronics and then accelerates the development of quantitative analysis in modern sports industry and athletes’ healthcare.

## Graphical abstract

Herein, the kernmantle structure is introduced into flexible electronics, specially, a robust and elastic kernmantle electronic braid (E‐braid) for self‐powered high‐impact sports monitoring is developed. This study introduces the new member—E‐braid for e‐textile family and then, accelerates the development of quantitative analysis in modern sports industry and athletes’ healthcare.

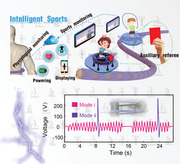

## 1 Introduction

In modern industry and daily life, the rope is an indispensable loading tool, and many fields require the participation of rope, including bridge construction,^[^ ^1^ ^]^ ropeway transportation,^[^ ^2^ ^]^ fall protection,^[^ ^3^ ^]^ and sports.^[^ ^4^ ^]^ Usually, ropes can be classified into dynamic ropes and static ropes according to their stretchability under load, and the stretch rate of static ropes is less than 5%. Higher elasticity allows a dynamic rope especially with a kernmantle construction to more gently absorb the energy of sudden high‐impacts, providing cushioning protection for the target user or machine. Though initially developed for rock climbing, kernmantle structure–braiding technique is an inherently large‐scale manufacturing strategy that produces multi‐functional braids with the integration of a broad range of materials, elasticity, and strength.^[^ ^5^ ^]^ However, the kernmantle structure has long been ignored in the field of flexible and wearable electronics for the following reasons: the lack of a proper electronic base unit can be designed into this structure; some essential materials for electronic devices often cannot be spun into yarns; unable to design reliable and reasonable conductive elements in the structure; and there exists a mismatch between the electronic units and braiding machine, which greatly limits their scalable production.

Yarn, one of the most significant inventions in modern garment industry, has become an indivisible component of our lives due to its unique properties such as easy to mass produce, light weight, nice wear‐comfort, and softness.^[^ ^6^, ^7^, ^8^ ^]^ Integrating yarns and electronic devices to further realize soft e‐textiles will bring brand new experiences to the community of flexible electronics while maintaining its original properties.^[^ ^9^, ^10^, ^11^, ^12^ ^]^ Human mechanical energy has been considered a potential green and sustainable energy solution for large‐scale distributed IoT devices.^[^ ^13^, ^14^, ^15^, ^16^, ^17^ ^]^ Yarn‐based devices can effectively convert large or small body motions into electrical energy,^[^ ^18^, ^19^, ^20^, ^21^, ^22^, ^23^ ^]^ which show great potential in building up around‐body interaction system. The significance of converting human‐mechanical energy into electricity motivates the exploration for cutting‐edge energy‐conversion technologies. Despite the numerous available working mechanisms the yarn‐based energy harvesters are mainly developed from such as electromagnetic effect^[^ ^24^, ^25^ ^]^ and piezoelectric effect,^[^ ^26^, ^27^ ^]^ an emerging triboelectric nanogenerator (TENG) utilizing the coupling effect of electrostatic‐induction and contact‐electrification has enabled widespread applications in low‐frequency mechanical energy harvesting and self‐powered sensing.^[^ ^28^, ^29^, ^30^ ^]^ With the advantages of light‐weight, easy implementation, and high output, TENG technique displays attractive possibility in the field of soft electronics, where large amounts of distributed monitoring devices must be applied.^[^ ^31^, ^32^, ^33^ ^]^ Benefiting from excellent scalability, high‐strength, wear‐comfort and light weight, yarn or yarn‐based TENG has attracted significant research interest in the energy and sensing community.^[^ ^34^, ^35^, ^36^ ^]^ However, current yarn‐based electronic devices tend to have low or even no elasticity, which greatly limits their practical employing scenarios where adapt‐to‐deformation is required.

In this study, a yarn‐based elastic E‐braid with kernmantle structure has been elaborately designed and we explored its potential in high‐impact sports monitoring. This method breaks through the low elastic characteristics of core‐spun‐yarn‐based electronic devices and further broadens its employing scenarios. Devices based on the E‐braids not only achieve high strength (31 Mpa), customized stretchability (depends on the selection of elastic core and density of weaving texture), and nice machine washability (>500 washes) but also exhibit excellent electrical stability (>200 000 cycles) in the stretched state. To evaluate the feasibility of the E‐braids in high‐impact flexible electronics, we successfully developed a self‐powered trampoline dual‐mode sensing system to continuously record the jumps and fouls at the same time, which could improve the efficiency of referees and also can provide guidance for athletes' daily training. Moreover, the E‐braids can be also used as wearable equipment to monitor the human respiration during sports. More importantly, different from the previous reported yarn‐based TENG devices which were manually wound onto other soft polymers^[^ ^37^ ^]^ or woven into plane fabrics by machine,^[^ ^38^, ^39^ ^]^ the fabricated TENG braids with kernmantle structure in this work perform a higher elasticity, better mechanical stability, and washability, providing solution for high‐impact sports monitoring, especially for large‐scale intelligent sports applications.

## 2 Results and Discussion

### 2.1 Fabrication of the E‐Braids

**Figure 1:**
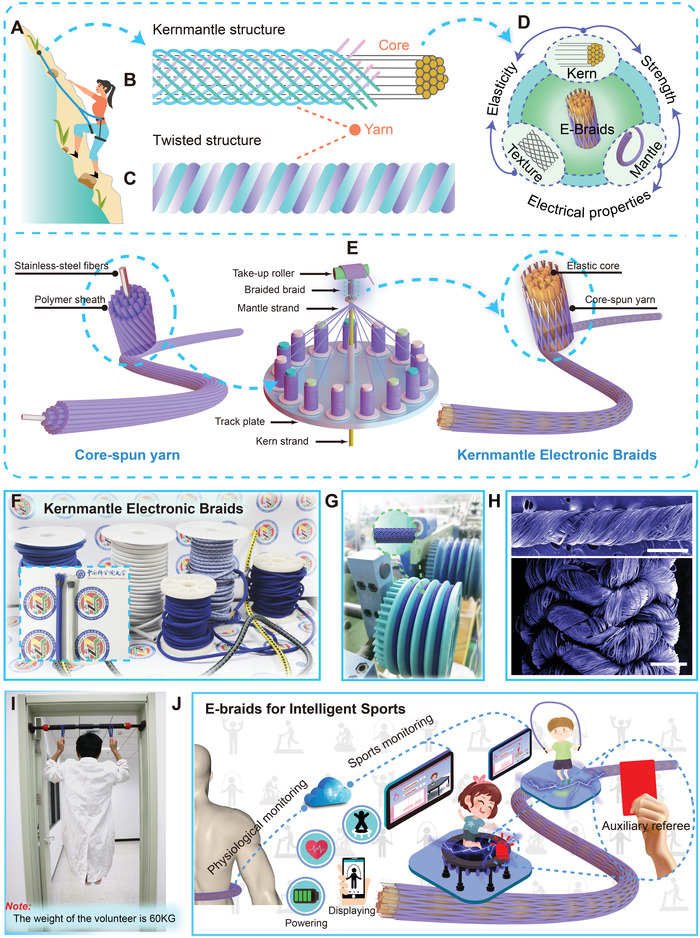
Structure illustration and fabrication of the electronic braids (E‐braids). A) Application of kernmantle structure ropes in the field of rock climbing. Typical structure diagram of B) kernmantle and C) twisted ropes. D) Contribution diagram of composition and structure to characteristic of the E‐braids. E) Schematic illustration showing the braiding process of the E‐braids. F) Optical image of all kinds of the E‐braids with different materials and strands (inset: enlarged photography of the two selected E‐braids). G) Photograph of the E‐braid on the braiding machine. The inserted photo in the top left is the enlarged view of the E‐braid. H) SEM images of a single core‐spun yarn (upper) and a 2 mm/8 strands E‐braid (lower), respectively (scale bar, 500 µm). I) Human weight bearing test of the E‐braid. J) Application outlook of the E‐braids in intelligent sports scene.

As we all know, the safety rope with a kernmantle structure is an indispensable protective equipment for enthusiasts of mountaineering and sports climbing (**Figure** **1**A). In practical applications, kernmantle and twisted construction are two commonly used rope structures for different occasions. Figure 1B,C demonstrates the typical structural differences between the two structures, which indicates that the kernmantle construction is structurally determined to possess higher extensibility, while the twisted structure exhibits only slight elongation under load. Aiming at ultrahigh strength, a certain elasticity, manufacturability, and electrical properties, an E‐braid with kernmantle construction was designed and fabricated through a continuous multiaxial braiding method. For demonstration, a contribution diagram of composition and structure to characteristic of the E‐braid is shown in Figure 1D. It can be seen that the elasticity of the E‐braid is mainly determined by the elastic kern and the woven texture of the mantle part. When it receives an axial stress, the outer texture will shrink, and the elastic core will further help the texture slowly recover when the external force is removed. The elasticity of the device as a whole is thus achieved. That is to say, we realized elastic E‐braids by using a composite structure composed of inelastic core‐spun yarn and elastic silicone fiber, which greatly broadens the application scenarios of stainless steel core‐spun yarns. Moreover, with regard to the mechanical strength of the E‐braid, both the kern and the mantle part are important contributors. Core‐spun yarns with core conductive fibers (stainless steel fibers) as electrodes and shell dielectric fibers (nylon fibers) as electrification layers are selected as the mantle strand, which endows the E‐braid with electrical properties. Here, we adopted the stainless steel fiber as the electrode material of the core‐spun yarns, which is an optimal solution based on our comprehensive consideration of cost and electrical conductivity. First of all, stainless steel fibers can be much cheaper than some other electrodes such as conductive carbon fibers. Second, although the resistance of stainless steel fibers may reach KΩ level after reaching a certain length (tens of meters), it is negligible compared to the triboelectric nanogenerator with an internal resistance of GΩ level. Note that the large‐scale fabrication process of core‐spun yarns has been reported in our previous study^[^ ^40^ ^]^ as illustrated in Figure S1, Supporting Information. Furthermore, multi‐strands of soft silicone fibers are adopted as the kern strand, owing to its excellent elasticity, inherent biocompatibility, high mechanical durability, and easy access. As demonstrated in Figure 1E, the fabrication and sketched structure of the E‐braid is introduced, where elastic rubber fibers as the inner core are tightly wound by the braided core‐spun yarns interweaving with each other. Consequently, with the assist of the multiaxial high‐speed cord braiding machine, hundreds of kilometers of E‐braids can be reliably obtained (Figure S2 and Video S1, Supporting Information). In practical applications, the actual parameters and materials of the inner elastomer and the outer core‐spun yarn all can be adjusted according to actual demands. For instance, different strands of elastic fibers can be selected to obtain varying degrees of elasticity, and the adopting strands number of core‐spun yarns can control the overall strength. As shown in Figure 1F, the optical images of all kinds of E‐braids are presented, including varied strands (8, 16, and 32), materials (PET and Nylon) and colors (white and blue) (Figure S3, Supporting Information). Hence, it is quite promising to realize large‐scale fabrication of customer‐designed E‐braids based on our universal designing strategy. The image of the E‐braids on the take‐up roller are shown in Figure 1G, and the insert is the enlarged view of the kernmantle braid. In order to clearly understand the structure of the electronic braids, scanning electron microscope images of a single core‐spun yarn (upper) and a 2 mm/8 strands E‐braid (lower) are shown in Figure 1H. Note that the 2 mm/8 strands means the diameter of the E‐braid is 2 mm and it consists of 8 strands of yarns. To visually demonstrate the advantage of high strength, a 60 kg adult can easily hang on the horizontal bar through an E‐braid for a long time as illustrated in Figure 1I. The versatility of this method and the excellent elasticity of the braiding structures will promote the application of this technology in the manufacture of various high‐strength intelligent electronic elastomers. The application prospect of the E‐braids in promoting intelligent sports is illustrated in Figure 1J.

### 2.2 Mechanical Properties Characterization

**Figure 2:**
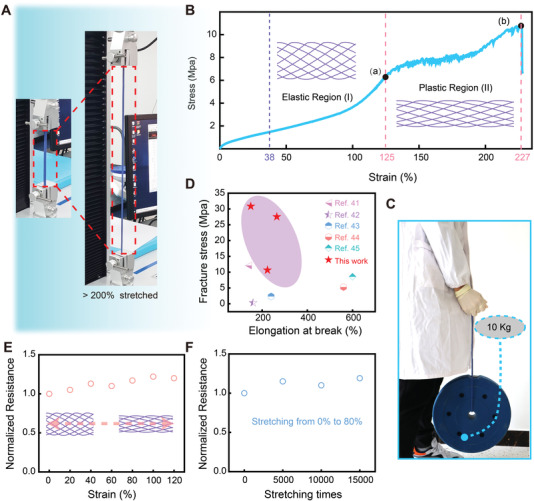
Mechanical characterization and resistance stability of the E‐braids during stretching. A) Photograph of the braid in a relaxed state and stretched by >200% on a stretching machine. B) Stress–strain curves of a 5.5 mm/32 strands E‐braid at a tensile rate of 50 mm min^−1^ (inset: texture changes before and after stretching). C) Optical image of the E‐braid lifting a 10 kg dumbbell. D) Comparison of the fracture stress and elongation at break of the E‐braids with other reported stretchable fiber/yarn‐based triboelectric devices. E) Resistance comparison of the E‐braids under different stretching conditions (0–120%). F) Characterization of resistance stability of the braid with different stretching times under 80% stretching rate. Sample Specification: 3 mm/16 strands, 5 cm in length.

As a sports monitoring solution for high strength and elastic occasions, it is necessary for us to investigate the mechanical characteristics of the E‐braids. First of all, the breaking stress of the E‐braids with various specifications and a single core‐spun yarn is presented in Table S1, Supporting Information. Considering that the follow‐up experiment requires a considerable impact test (60 kg adult jumping), we choose a 5.5 mm/32 strand E‐braid with the largest breaking force as the typical research object. The optical images of the E‐braid (5.5 mm/32 strands) at 0% stretch and >200% stretch are shown in **Figure** **2**A. The typical stress–strain curve of the E‐braid is displayed in Figure 2B, which exhibits nonlinear elastic behavior within the low extension region (0% to 125%) and plastic deformation under higher extension (125% to 227%). It is obvious that the tensile curve of the E‐braid in the plastic region appeared with intermittent yield, which was different from conventional elastomers. We analyzed that the reason for this situation is related to mismatch of elastic constant of kern fibers and mantle yarns. When the braid was further stretched, it got damaged at an ultimate tensile strength of as high as 256.4 N (≈10.8 Mpa) as depicted in Figure S4, Supporting Information. In addition, the stress–strain curves of other two E‐braids are shown in Figure S5, Supporting Information. Compared with traditional elastomers, such as pure silicone rubber and pure TPU, E‐braids with the help of yarns, have achieved higher strength despite making certain sacrifices in elastic. Similarly, the electronic braids possess much higher elasticity than that of a single core‐spun yarn, which was destroyed quickly when it was stretched ≈38% at a breakage force of ≈20 N (Figure S6, Supporting Information). The interwoven structure in the braid makes it have heterogeneous extension properties in the two distinct regions: the extension of the texture (region I) and the extension of the yarn itself (region II). When the braid is stretched, the mantle yarns will be stretched slightly while the weaving gaps are enlarged more significantly. The outer interwoven texture allows the E‐braid to have space to be stretched under stress, while the inner elastomer core allows it to naturally rebound after the external force is removed. The texture photographs of the E‐braid during elastic stretching and recovery are illustrated in Figure S7, Supporting Information, which displays the excellent elastic ability. For demonstration, the optical photos of E‐braids with different elasticity before and after stretching are shown in Figure S8 and Figure S9, Supporting Information, respectively. The above results successfully confirmed that the combination of core‐spun yarns and elastic rubber fibers through kernmantle structure perfectly compensates for their shortcomings and meets the requirements of ultra‐high strength and high elastic. Furthermore, Figure 2C shows the optical photo of lifting a 10 kg dumbbell with a single E‐braid. As shown in Figure 2D; Table S2, Supporting Information, we compared the electrical performance of the present E‐braids with the currently reported stretchable fiber/yarn based triboelectric devices on the basis of fracture stress and stretchability.^[^ ^41^, ^42^, ^43^, ^44^, ^45^ ^]^ The E‐braids show comparatively excellent performance in terms of mechanical strength toward highly conductive, superelastic, and high‐strength braiding triboelectric elastomers. Next, the conductive stability under tensile of the fabricated braid is evaluated. First, the resistance stability of E‐braids under different tensile conditions is demonstrated in Figure 2E, where the resistance change of the E‐braid is almost negligible under different stretches in the range of 0% to 120%. In addition, Figure 2F illustrates that the E‐braid can show excellent resistance stability for 15 000 cycles under 80% stretch. These results further confirmed the satisfactory robustness and mechanical stability of the E‐braids, which indicated that they have great potential for applications where high deformation is required.

### 2.3 Basic Working Mechanism and Performance Analysis

Benefitting from adopting core‐spun yarns as the mantle part, the E‐braids can easily convert human mechanical energy into electric energy/signal via an emerging triboelectric nanogenerator technology. Taking FEP as the moving object, the working mechanism of the E‐braids in single‐electrode mode is illustrated in Figure S10A, Supporting Information. When the E‐braid is in contact with other materials, triboelectric charge will be generated on their surface and then an electrical field will be built between two friction surfaces. According to the triboelectric series, the nylon has a strong ability to gain electrons and the FEP tends to lose electrons.^[^ ^46^ ^]^ The periodic contact/separation of the two tribolayers will generate pulse electron flows between the respective electrodes based on Maxwell's displacement current.^[^ ^47^ ^]^ The potential distribution of the E‐braids was simulated by COMSOL for better understanding the operating mechanisms, as shown in Figure S10B, Supporting Information. In the braiding process of E‐braids, the number of winding yarns and the specification of the elastic core are two important parameters, which determine the effective friction area. Next, their influence on the triboelectric output is investigated. As illustrated in Figure S11A, Supporting Information, the electrical outputs of the E‐braids with different strands are compared, which are almost free from the influence. This result may be attributed to the fact that the number of winding yarns hasn't made a contribution to the increased contact area because the diameter of the core‐spun yarns is fixed. In addition, the electrical output of 32 strands E braid with different diameters was also discussed, as demonstrated in Figure S11B, Supporting Information. The result shows that the electrical output demonstrates a slight upward trend with the increase of diameter, which can be attributed to the rising of the contact area. Comprehensively considering the factors of strength and electrical output, our follow‐up test mainly adopted the E‐braids with a specification of 5.5 mm/32 strands.

**Figure 3:**
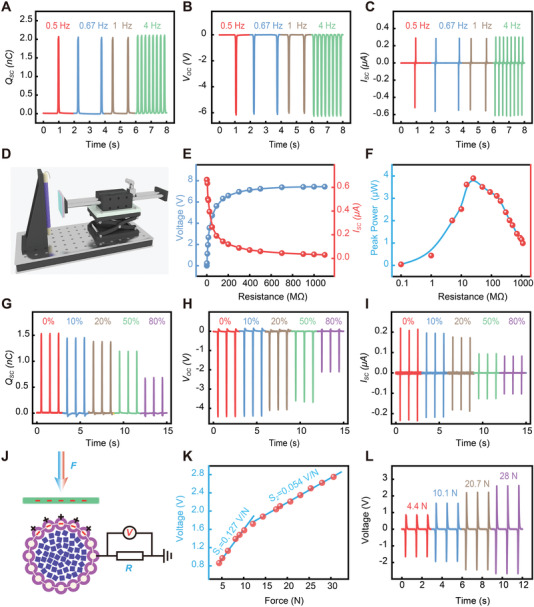
Output performance of the E‐braid (3 mm/16 strands, length:5 cm). A–C) Electrical output performance of the E‐braids under various frequencies (0.5–4 Hz), including *Q_SC_* (A), *V_OC_* (B), and *I*_SC_ (C). D) Diagram illustration of the test platform. E) Dependence of current density and voltage of the E‐braids on the external load resistance with a tapping frequency of 1 Hz. F) Variation of power density of the E‐braids as a function of load resistance. G–I) Electrical output performance of the E‐braids at different stretch ratios (0–80%), including *Q*_SC_ (G), *V*_OC_ (H), and *I*_SC_ (I). J) Schematic illustration of the E‐braids and the measured mode of electrical signals with a 100 MΩ load resistance. K) The output voltage as a function of applied force. L) Output voltage signals of the TENG under different force.

Furthermore, some conventional electrical properties of the E‐braids have been comprehensively studied to evaluate its sensing ability. First of all, the frequency‐dependent electrical output of the E‐braids, including short‐circuit charge transfer (*Q*_SC_), open‐circuit voltage (*V*_OC_), and short‐circuit current (*I*_SC_) is characterized, as shown in **Figure** **3**A–C. As the frequency of contact separation increased from 1 to 4 Hz, the peak *Q*_SC_ and peak V_OC_ increased a little, and the peak *I*_SC_ increased slightly. These results are in accordance with the fact that the contact force is fixed and dt in formula (*I*_SC_ = dQ/dt) is compressed with increased frequency. The electrical output performance was measured based on an integrated test platform, including a high‐resistance electrometer and a data acquisition card for signal acquisition, and a linear motor for periodic movement generating (Figure 3D). For demonstration purpose, we studied the dependence of current density and voltage of the E‐braids on the external load resistance with an operating frequency of 1 Hz, as shown in Figure 3E. The curves illustrated that the load voltage kept increasing with the increase of resistance while the current density demonstrated an opposite trend, which was in line with the basic laws of electricity. Moreover, the output power of the E‐braids was calculated as *I* ^2^ × *R* by measuring its output current under various loading resistance ranges from 0.1 MΩ to 1GΩ (Figure 3F). A peak power of 3.9 µW can be achieved at an external load of 25 MΩ. As we all know, the stability and washability of yarn‐based electronic devices has always been a thorny problem, which determines its viability in real‐life application. As shown in Figure S12, Supporting Information, the output performance of the E‐braid before and after machine washing (>500 cycles) is studied. The results demonstrate that its electrical properties have not been significantly attenuated during washing, which indicates that it has excellent machine washability. It is worth mentioning that such good stability and washability are highly related to our use of materials that are completely close to commercial clothing. As a stretchable device, it is necessary to characterize its electrical properties under tension condition. Therefore, we tested the electrical output of E‐braids in the range of 0% to 80% stretch, including *Q*_SC_, *V*_OC_, and *I*_SC_. As demonstrated in Figure 3G–I, the electrical output of E‐braids has shown a certain downward trend with the increase of stretch rate, which can be attributed to the effective contact area becoming smaller as it stretches. For efficient sports monitoring, the E‐braids should be sensitive to the externally applied force. In addition to the foregoing characterization of tensile stability, washability, and mechanical stability, here we evaluated the sensitivity of the E‐braids‐based sensor. The basic testing principle was demonstrated in Figure 3J, where the resistance of the load was 100MΩ. As shown in Figure 3K, the E‐braids‐based sensor exhibited an excellent linear response with a force sensitivity of 0.127 V N^−1^ in low pressure region, which was mainly attributed to the increase of the effective contact area. While at force region of greater than 12 N, the force sensitivity was decreased to 0.054 V N^−1^ due to the limited growth of the contact area as the force increased. For demonstration, Figure 3L demonstrates the output voltage of the E‐braids under several applied forces, further showing that the output performance increases with the applied force.

### 2.4 Self‐Powered Trampoline Sport Dual‐Mode Sensing System Based on E‐Braids

**Figure 4:**
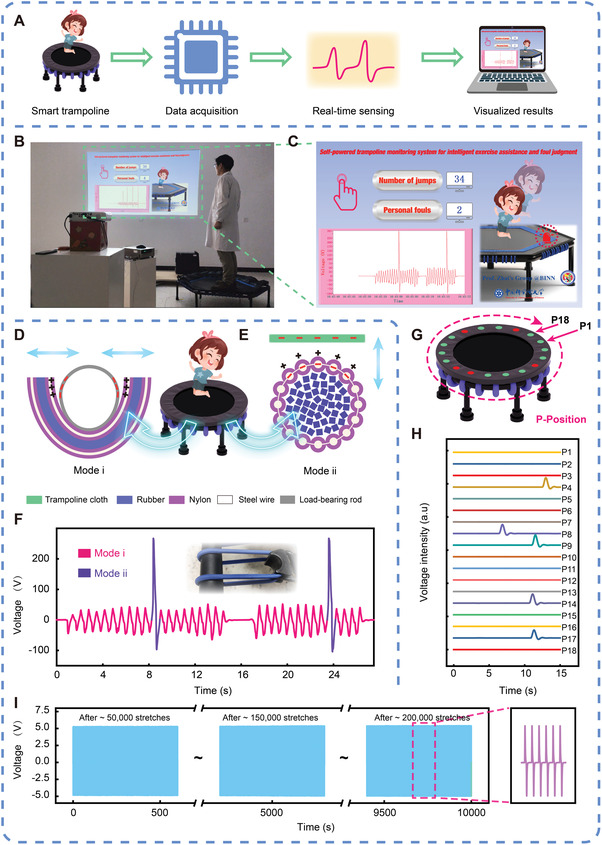
Application of the E‐braids in a self‐powered trampoline dual‐mode sensing system (Sample Specification: 5 mm/32 strands). A) Scheme diagram of the E‐braids based self‐powered trampoline dual‐mode sensing system. B) Photograph and C) screenshot of the real‐time counting/judgement result showing a man jumping on the trampoline. Working principle of the E‐braids at D) jumping counting mode and E) fouls counting mode. F) The real‐time signal recording of the dual‐mode sensing system. The inset was an optical image of an E‐braid installed on the trampoline. G) Demonstration of the self‐powered foul position statistical system. H) Real‐time output voltage signals when a man stepped on the edge of the E‐braids array‐based trampoline. I) Electrical properties of E‐braids subjected to ≈200 000 stretches (inset: enlarged view of the selected region).

Trampoline gymnastics, as one of the most popular events of Olympic Games, is very pervasive in both amateur sports and professional competitions. Improving the intelligence of the trampoline is of great significance for scientific exercising and promoting the efficiency of athletes/referees. In the rules of modern Olympic trampoline, there is a penalty point when the athlete touches the spring. In fact, this deduction point is mainly captured by the referee's visual judgment, which can easily lead to a misjudgment. Herein, a self‐powered trampoline dual‐mode sensing system for jumping counting and fouls monitoring is constructed based on the fabricated E‐braids. The detailed assembling process is illustrated in the Experimental Section and some related optical images are shown in Figure S13, Supporting Information. As demonstrated in **Figure** **4**A, when a person is jumping on the trampoline, the E‐braid will generate an output signal. Through a high‐speed data acquisition module, real‐time sensing signals will be detected and fed back to the computer. After signal processing, the visualized results can be displayed in the system. Figure 4B illustrates the actual operating images of the self‐powered dual‐mode sensing system. The screenshot of the E‐braids‐based sensing system is shown in Figure 4C, including real‐time sensing signals displaying, statistics of jumps and fouls, and athletes jumping imitation. Next, the operation mode and working mechanism of the trampoline dual‐mode sensing system are demonstrated in detail, as described in Figure 4D,E. When a person is jumping on the trampoline, the E‐braid will generate a low voltage output (signal i) due to the back and forth contact‐separation during the load‐bearing rod and the braid, and the trampoline monitoring system works in mode i (jumping counting mode). As there is a one‐to‐one relationship between the signal i and the number of jumps, mode i can be used for training counting. When the person steps on the trampoline spring (E‐braid) during jumping, the E‐braid will generate a relatively high voltage output (signal ii) due to the contact‐separation between the braid and trampoline cloth, and the trampoline monitoring system will work in mode ii (fouls monitoring mode). In actual games, mode ii can be applied to assist referees in judging fouls involving stepping on the spring. Video S2, Supporting Information, demonstrates the actual operation scheme of the trampoline monitoring system. As illustrated in Figure 4F, the peak intensity of signal ii can reach 5–15 times that of signal i, so as to achieve good discrimination and indicating that the E‐braids‐based trampoline sensing system can work precisely and stably in dual mode. It is worth noting that the main reason for the obvious difference in the output signals of the two modes is the effective contact separation area. To improve the athletic training efficiency and umpires’ refereeing accuracy, a self‐powered foul position statistical system is built based on the E‐braids array in Figure 4G. When athletes step on different edge positions, their foul information will be further recorded. The multi‐channel output voltage signals of the system are shown in Figure 4H, indicating the feasibility of our sensing system for foul position tracing. In order to be used for long‐term and stable trampoline monitoring, the deformation of the E‐braids in this study should generally be controlled below 100%. In addition, the electrical stability of the E‐braids has been investigated under ≈200 000 strengthening stretches (80%) at a load resistance of 100 MΩ and the signal indicates that the output voltage has always been stable, as shown in Figure 4I, which further verifies that our E‐braids can achieve stable and reliable high‐intensity intelligent sports monitoring.

### 2.5 Other Application Cases of the E‐Braids

**Figure 5:**
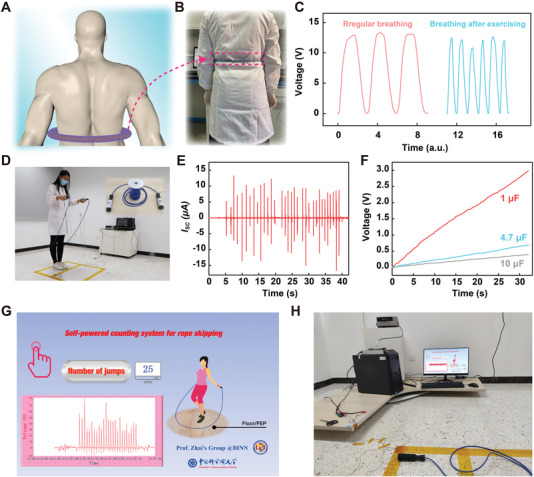
E‐braids for wearable physiological monitoring and energy harvesting during exercising (Sample Specification: 5 mm/32 strands). A) Schematic illustration and B) photograph of the E‐braids worn around the torso for respiration monitoring. C) The generated signals of the breathing monitoring system under different breathing state. D–F) The E‐braids for exercising energy harvesting: optical image of the energy harvesting system (D), electrical signals generated by rope skipping (E), and charging curve of different capacitors by rope skipping (F). G,H) E‐braids for self‐powered skipping counting system: screenshot showing the real‐time sensing system of the skipping counting system (G), photograph of the self‐powered skipping counting system (H).

To verify the versatility of the E‐braids, some other applications have been investigated as well. First, we design a human abdominal breathing monitoring system by placing the E‐braids in a silicone tube, as demonstrated in **Figure** **5**A,B. When a person is breathing, regular electrical signals will be detected by the contact separation of the E‐braids and the inner wall of silicone tube. Considering to shield the static electricity from the outside and the human body, the silicone tube with a thickness of up to 150 µm was adopted. Figure 5C illustrates the breathing signals of the same person when calm and after exercise, showing that the breathing rate was significantly faster after exercise. This indicates that our E‐braid can be used for both sports monitoring and physiological signal monitoring in intelligent sports. Then, the E‐braids are designed as a skipping rope for sports energy capturing or counting sensing (Figure S14, Supporting Information). In order to improve the electrical output, we laid a layer of FEP film with high triboelectricity on the floor (Figure 5D). When the E‐braids‐based skipping rope acts as an energy harvester, the continuous kinetic energy can be collected and stored during the jump. In Figure 5E, the output current of the E‐braids by jumping was measured and the electric energy could be stored in commercial capacitors through a facile power management circuit (Figure S15, Supporting Information). In addition, the capabilities of the E‐braids‐based skipping rope for charging different capacitors were also studied, as presented in Figure 5F. The voltage of the capacitor of 1 µF can be raised to 3 V within ≈30 s by jumping with the skipping rope. When the E‐braids‐based skipping rope acts as a counting sensor, as illustrated in Figure 5G,H, the number of skips can be recorded for exercise guidance. Furthermore, Figure S16, Supporting Information, shows a data flow chart for an ideal system for human physiological monitoring by self‐powered E‐braids‐based technologies. In the system, the E‐braids‐based device provides both the power source and the sensing solution.

## 3 Conclusion

In summary, we successfully present ultrahigh‐strength and high‐elastic kernmantle‐structure–electronic braids by winding/weaving core‐spun yarns with conventional elastic rubber/yarns to form high‐performance tribo‐sensor toward high‐impact intelligent sports. Our strategy is facile and can be well compatible with the current textile industry process. Traditional non‐elastic yarns have achieved high elastic electronic devices through a synergistic effect of texture design and kern elastomer guidance. Without changing the original functions of the trampoline, the E‐braids have been successfully applied to the trampoline sports counting/foul monitoring dual‐mode real‐time sensing system. Moreover, the E‐braids could also collect biomechanical energy such as rope skipping for mobile electronic devices. We expect these kernmantle‐structure “electronic braids,” which are beyond the conventional elastic stretching purpose, to pave the way, not only limited to the field of intelligent sports but also for self‐powered elastic electronics.

## 4 Experimental Section

### Fabrication of Core–Spun Yarns and Electronic Braids

The core‐spun yarns were obtained through a commercial high‐speed spinning frame and the detailed fabrication process has been described in the authors’ previous work.^[^ ^40^, ^48^ ^]^ Using the core‐spun yarn obtained above as the basic unit and high‐elastic silicone as the core, large‐scale electronic braids can be obtained through commercial braiding machines.

### Fabrication of the E‐Braids‐Based Trampoline Dual‐Mode Sensing Unit

First, 50 cm 5.5 mm/32 strands E‐braids were prepared and the electrodes part of each core‐spun yarn was connected in series. Considering that there would be displacement occurring between the rubber core and the yarn shell during repetitive stretching, the two ends of the E‐braids were then fixed through a type 8 double hole aluminum ferrule and reinforced with a hammer. Last, the sealed and reinforced E‐braids were installed on the trampoline and a self‐powered trampoline dual‐mode sensing unit was constructed successfully.

### Characterization and Measurements

Field emission scanning electron microscope (Nova NanoSEM 450) was used to characterize the morphologies of the core‐spun yarn and the electronic braids. A linear motor (B01‐37 × 166/260, LinMot) was used to simulate regular motions. The transferred charges, open‐circuit voltage, and short‐circuit current were measured by a programmable electrometer (Keithley model 6517B). A digital display force gauge (HP‐50, HANDPI) was used to detect the applied force. The mechanical tensile properties were measured by a tensile mechanical testing system (YL‐S90, Yuelian Testing Machines Co., Ltd, Dongguan, China). A voltage acquisition card (NI PCI‐6259) was used to further display the signal collected by the electrometer in the computer. A multi‐channel acquisition card (NI USB‐6349) was used for 18‐channel voltage acquisition in the foul position sensing system. All the data acquisition and processing software were based on lab view in this study. The changes in resistance of the devices were detected by a Keithley electrometer 2450.

### Statistical Analysis

The values of resistance data in Figure 2E,F were normalized. The statistical analysis was performed using OriginPro 2021b Learning Edition (Origin‐Lab Corporation).

## Conflict of Interest

The authors declare no conflict of interest.

## Author Contributions

W.W. and A.Y. contributed equally to this work. *Generated the design concept*: Z.L.W., J.Y.Z., and W.W. *Designed and performed the experiment*: W.W. and A.Y. *Participated in the data discussion*: Y.W., M.J., P. G., L.R., and D.G. *Helpful comments to the manuscript*: X.P. *Analyzed the data and prepared the figures*: W.W., A.Y., and J.Z. *Wrote and revised the manuscript*: W.W., A.Y., J.Z., and Z.L.W.

## Supporting information

Supporting Information

Supplemental Video 1

Supplemental Video 2

## Data Availability

The data that support the findings of this study are available from the corresponding author upon reasonable request.
