# Supplementary material for: Elastic Kernmantle E‐Braids for High‐Impact Sports Monitoring
Source: Adv Sci (Weinh). 2022 Jun 27;9(25):2202489. doi: 10.1002/advs.202202489 (PMC9443433; doi:10.1002/advs.202202489)
Supplement: Supplementary file 1 — Supporting Information [file ADVS-9-2202489-s002.pdf]

Supporting Information

# Elastic kernmantle E-braids for high-impact sports monitoring

*Wei Wang, Aifang Yu, Yulong Wang, Mengmeng Jia, Pengwen Guo, Lele Ren, Di Guo, Xiong Pu, Zhong Lin Wang*, Junyi Zhai**

***Corresponding Author.** Email: zhong.wang@mse.gatech.edu(Z.L.W.); jyzhai@binn.cas.cn (J.Z.)

**This PDF file includes:**

Figures S1 to S16

Tables S1 to S2

Legends for Videos S1 to S2

**Supplementary Figures**

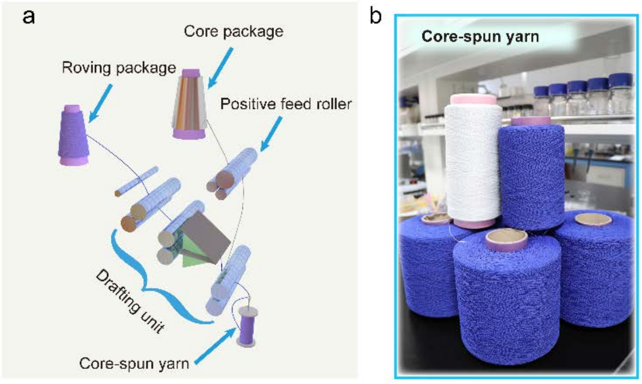


**Figure S1 | Fabrication and photograph of the basic units: core-spun yarns. a** Diagram illustration showing the manufacture of the core-spun yarns. **b** Optical image of the core-spun yarns.

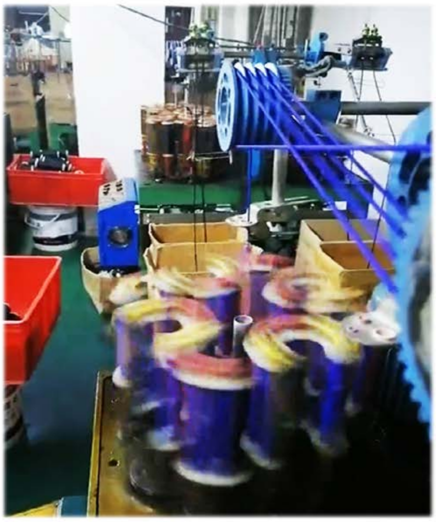

**Figure S2 | Photograph of the working process of the multiaxial high speed cord braiding machine.**

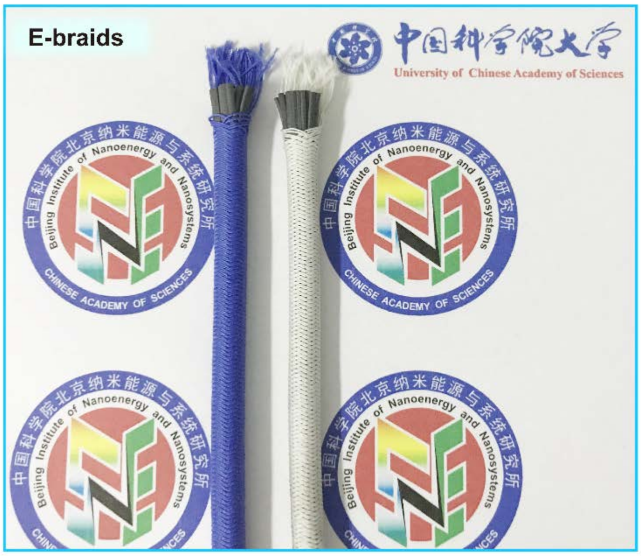


**Figure S3 | Enlarged view of E-braids in two different materials (left: Nylon; right: PET).**

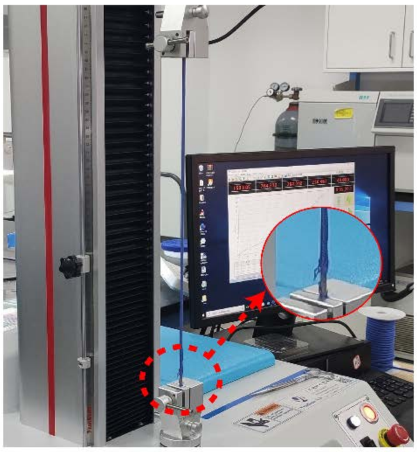

**Figure S4 | Photograph of the broken E-braids.**

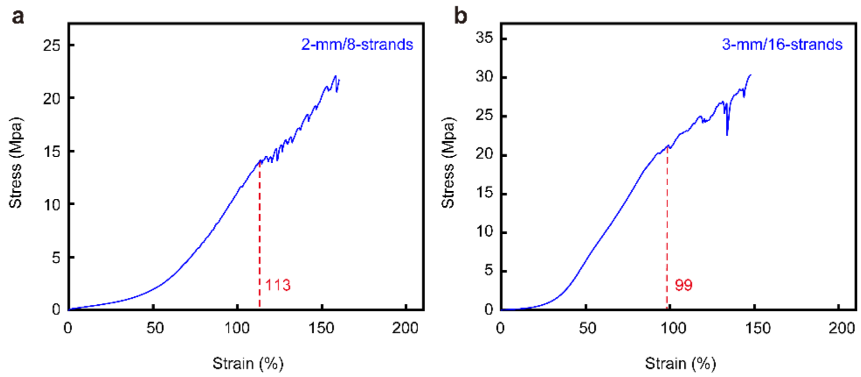


**Figure S5 | Stress-strain curves of other two E-braids at a tensile rate of 50 mm min$^{-1}$. a** 2-mm/8-strands. **b** 3-mm/16-strands.

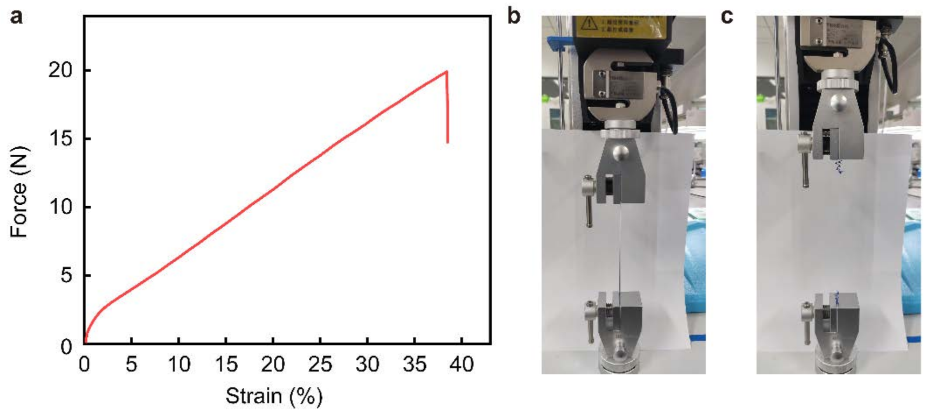


**Figure S6 | Mechanical properties of a single core-spun yarn. a** Tensile force–strain curves of a single yarn. **b** Photograph of a single core-spun yarn in a relaxed state. **c** Photograph of the broken core-spun yarn.

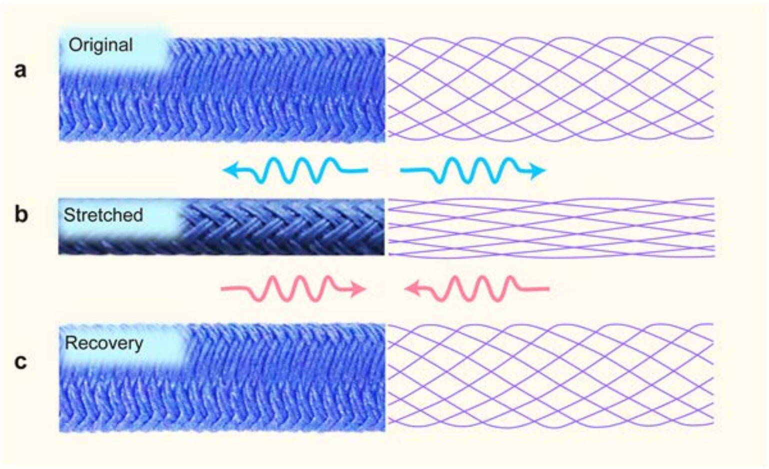


**Figure S7 | Texture photographs comparison of the E-braid during elastic stretching and recovery. a** Original. **b** Stretched. **c** Recovery.

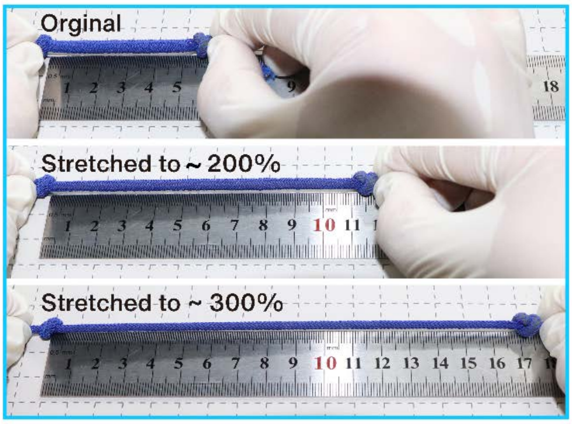


**Figure S8 | Photography of a single core E-braid stretched to ~300% of its original length.**

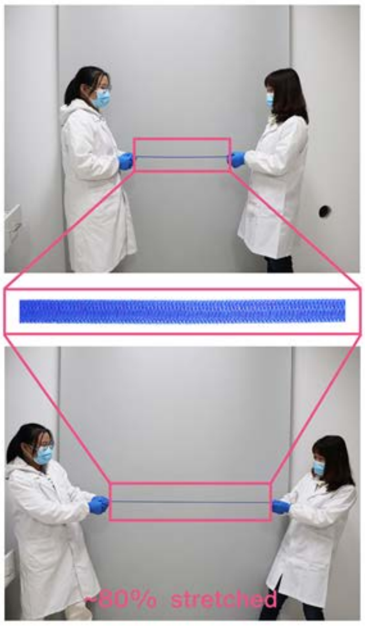


**Figure S9 | Optical images of two people stretching the high-strength E-braid by 80%.**

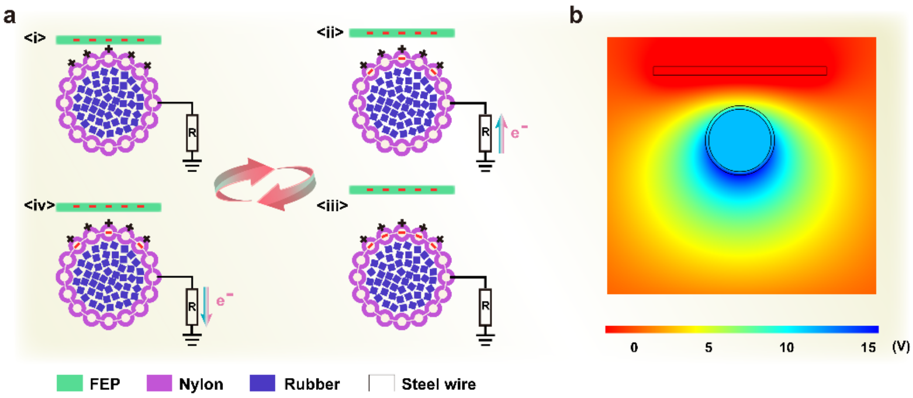


**Figure S10 | Working mechanism of the E-braids. a** Schematics of the operating principle. **b** Potential simulation by COMSOL.

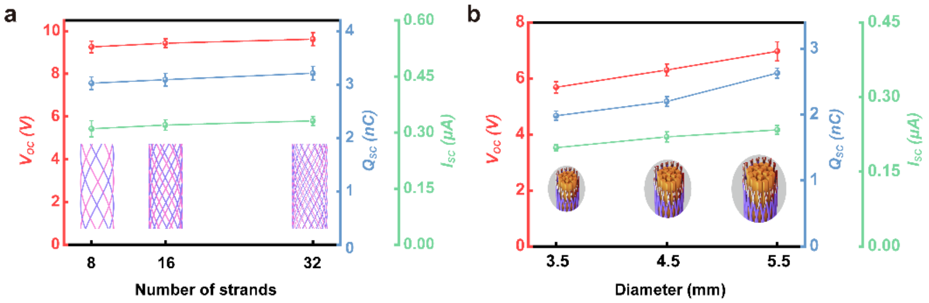


**Figure S11 | Electrical performance analysis of the E-braids. a** Influence of the number of strands on electrical performance. **b** Diameter-dependent electrical output of the E-braids.

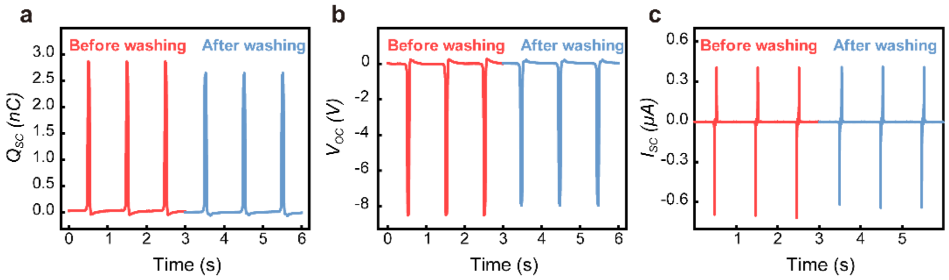


**Figure S12 | Electric properties of the E-braids washed up to 500 cycles under machine washing, including a** *Qsc*, **b** *Voc*, **c** *Isc*. Sample Specification: 5-mm/32-strands, 5mm in length.

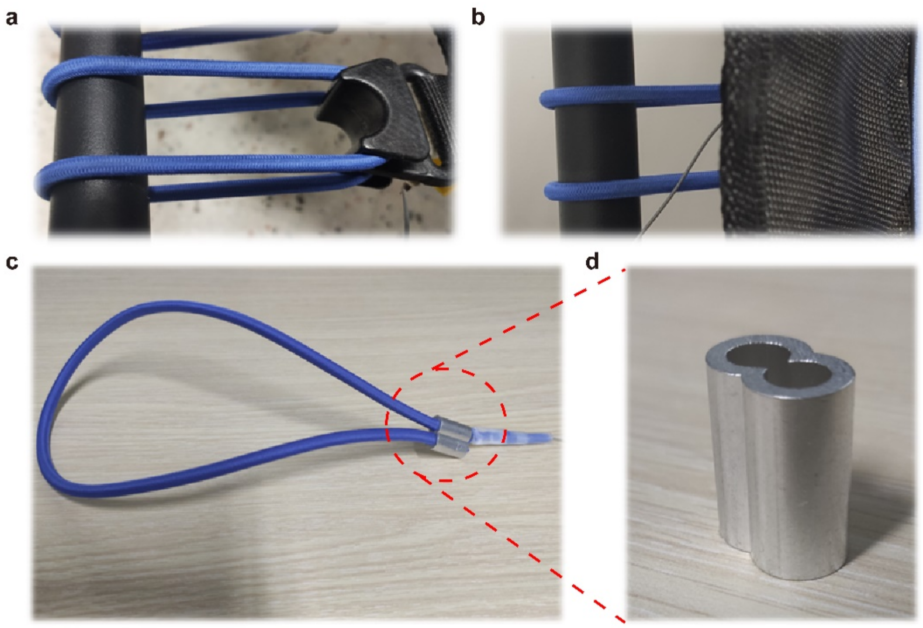


**Figure S13 | Photograph of the E-braid in the self-powered trampoline dual-mode sensing system. a-b** Photograph of the E-braids installed on the trampoline. **c** Optical photo of the fixed electronic braids. **d** Photograph of the type 8 double hole aluminum ferrule.

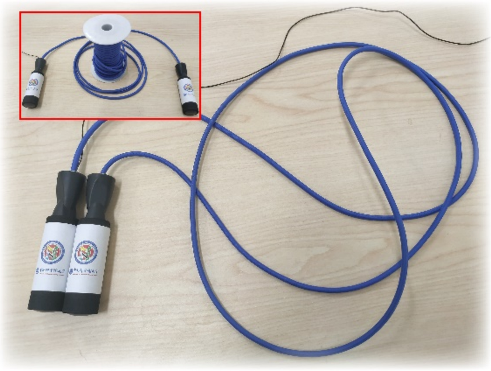

**Figure S14 | Photograph of the E-braids based triboelectric energy harvester.**

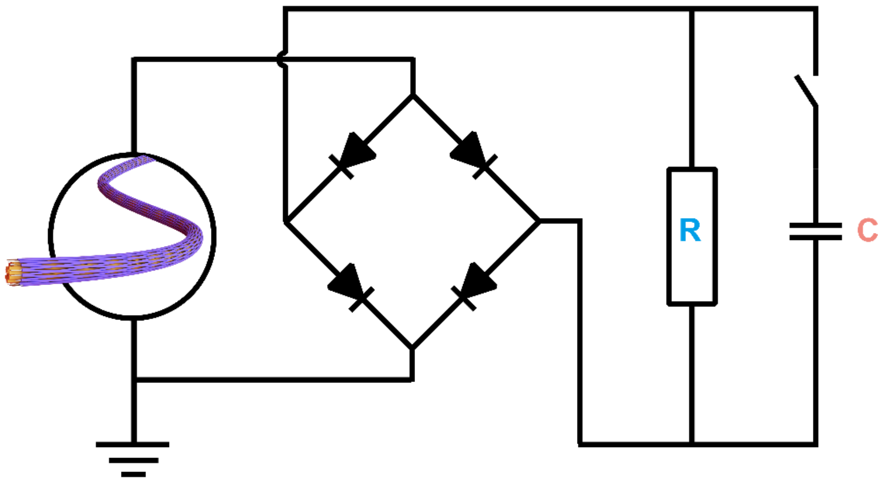


**Figure S15 | Circuit diagram of the E-braid based energy harvesting system.**

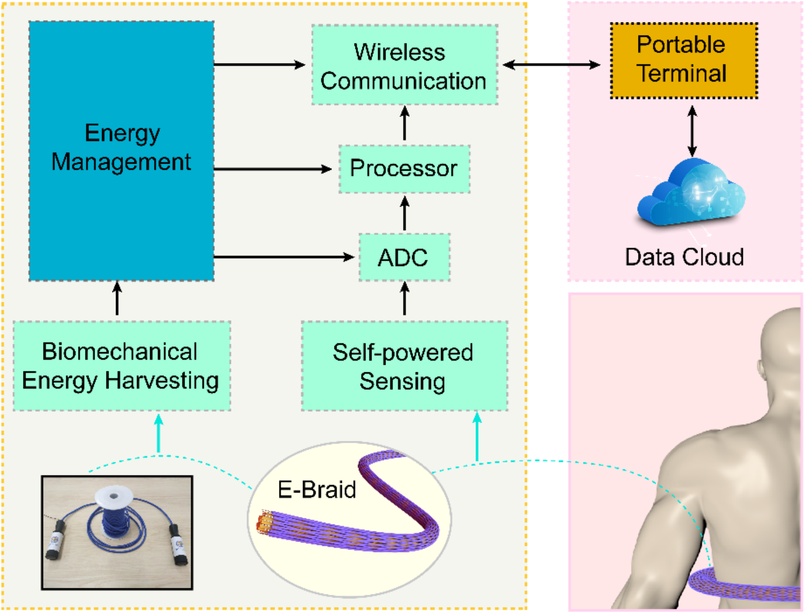


**Figure S16 | Data flow chart for an ideal system for human physiological monitoring by self-powered E-braids based technologies.**

## Supplementary Tables

**Table S1 | The breaking stress of E-braids with different specifications.**

| Sample specifications | Breaking stress (Mpa) | Elastic region(%) | Elongation at break (%) |
|---|---|---|---|
| A single core-spun yarn | — | No-elasticity | 38 |
| A 2-mm/8-strands braid | 27.6 | 0-113 | 265 |
| A 3-mm/16-strands braid | 31 | 0-99 | 150 |
| A 5.5-mm/32-strands braid | 10.8 | 0-125 | 227 |

**Table S2 | Comparison of the fracture stress and elongation at break of the E-braids with other reported stretchable fibre/yarn based triboelectric devices.**

| Structure | Electrode | Elongation at break (%) | Fracture stress （Mpa） | Output *Voc* (V) | Ref. |
|---|---|---|---|---|---|
| Fibre-structured | MXene/CNT/PU | 140 | 12 | 6.5 | 41 |
| Yarn embedded | Silver coated nylon | 160 | 0.45 | 160(Textile) | 42 |
| Core–sheath yarn | Silver coated nylon | 240 | 2.24 | 19 | 43 |
| Microstructured fibre | Liquid metal | 560 | 5.2 | 5.5 (3cm) | 44 |
| Sheath-core | Metal fiber | 600 | 8.5 | 4.16 | 45 |
| Kernmantle braiding | Stainless steel | 150 | 31 | — | **This work** |
| | | 227 | 10.8 | — | |
| | | 265 | 27.6 | — | |

**Video S1.**

Fabrication of the electronic braids (E-braids).

**Video S2.**

Demonstration of the of the trampoline dule-mode sensing system.
